# Joint kinematic responses of Olympic medallist skiers to repeated slalom runs

**DOI:** 10.1113/EP091743

**Published:** 2024-09-05

**Authors:** Marine Alhammoud, Robin Trama, Christophe André Hautier, Olivier Girard, Sébastien Racinais, Clint Hansen, Frédéric Meyer, Abdulaziz Farooq, Jérémy Coint, Thibaut Trameau, Loïc Brun, Baptiste Morel

**Affiliations:** ^1^ Inter‐University Laboratory of Human Movement Biology Universite Claude Bernard Lyon 1 Villeurbanne France; ^2^ Human Performance Laboratory, Faculty of Kinesiology University of Calgary Calgary Alberta Canada; ^3^ School of Human Sciences (Exercise and Sport Science) The University of Western Australia Crawley Western Australia Australia; ^4^ Environnement Stress Unit CREPS Montpellier Font‐Romeu Montpellier France; ^5^ Department of Neurology Christian‐Albrechts‐Universität zu Kiel Medizinische Fakultat Kiel Germany; ^6^ Digital Signal Processing Group, Department of Informatics University of Oslo Oslo Norway; ^7^ FIFA Center Aspetar ‐ Orthopaedic and Sports Medicine Hospital Doha Qatar; ^8^ French Ski Federation Annecy France; ^9^ Inter‐University Laboratory of Human Movement Biology Savoie Mont Blanc University Chambéry France

**Keywords:** fatigue, multiple kinematic patterns, performance stability, winter sports

## Abstract

This case study aims to examine changes in the lower limb joint kinematic profile and performance stability induced by repeated ski runs in two world‐class alpine skiers. Two Olympic medallist alpine skiers were tested during their slalom training, with continuous recording of right knee and hip angles, along with turn time and run time. The eight runs of the training session were analysed with linear mixed models. Results showed no effect of runs repetition on performance (i.e., run and turn time; *P* ≥ 0.279). There was no global effect of runs repetition on minimal and maximal angles for either the knee or the hip (*P *> 0.151). There was an interaction between run and leg for the maximal angle of both the knee and hip (*P* ≤ 0.047), which increased across runs for the outside leg and decreased for the inside leg. The maximal angular velocity for both the knee and hip increased with runs repetition in extension (*P* ≤ 0.028). There were no overall changes in maximal angular velocity in flexion with runs repetition (*P* ≥ 0.264), but there was an interaction between run and leg for the knee (*P *< 0.001) due to faster eccentric velocities across runs for the outside leg and slower velocities for the inside leg. In conclusion, the observed joint kinematic alterations without concomitant performance impairment support the concept of multiple movement strategies in athletes to achieve similar performance, especially under fatigue conditions.

## Abstract (highlights)

- **What is the main observation in this case?**
  Joint kinematics showed minor alterations across a training session as world‐class skiers performed eight slaloms.
- **What insights does it reveal?**
  In the absence of changes in turn and run time, these alterations should be interpreted as adaptations in skiing style rather than a shift towards a less functional skiing technique. Some of these adaptations support the concept of multiple movement strategies in athletes to achieve similar performance, especially under fatigue conditions.

## 1 INTRODUCTION

Alpine skiing, a winter Olympic sport since 1936, includes downhill, super‐giant slalom, giant slalom, slalom (SL), alpine combined (downhill and slalom), and parallel slalom. Among those disciplines, SL is characterized by highly dynamic movements with higher knee flexion/extension velocities and lower isometric duration than other alpine skiing disciplines (Alhammoud et al., 2020; Berg & Eiken, 1999; Kröll et al., 2015). A pilot study indicated that SL was the only discipline showing kinematic alterations across four repeated runs in elite skiers (Alhammoud et al., 2022). While this previous study was limited to four runs and included data from various athletes, disciplines and days, it prompted the question of the kinematic variations during a SL training session in World Cup skiers, typically including 6–12 runs (Gilgien et al., 2018). The repetition of turns represents a considerable neuromuscular demand (Turnbull et al., 2009) potentially leading to fatigue‐related impairments as well as muscle damage (Enoka & Duchateau, 2016). For example, both completion time and rate of incomplete runs (due to a disqualifying fault or a missed turning pole) have been reported to increase across eight training runs (White & Wells, 2015). On one hand, kinematic impairments could be significant, potentially causing performance decrements (Spörri et al., 2012, 2018) and technical errors associated to injury pattern such as dynamic snowplow, sliding sideways and slip‐catch mechanisms (Bere et al., 2011; Spörri et al., 2022). On the other hand, studies have shown the possibility to maintain performance during jumps while adapting the kinematic pattern to fatigue (Gathercole et al., 2015a; Ruggiero et al., 2022). In the context of skiing, kinematic alterations likely depend on skiers’ level (Alhammoud et al., 2022; Kiryu et al., 2011; Kröll et al., 2011).

Therefore, the aim of this study was to determine the performance and joint kinematic alterations in two Olympic medallists performing a typical training session (i.e., eight SL runs). We hypothesized that the athlete's performance would be minimally affected with runs repetition, despite joint kinematic evolution during the session.

## 2 METHODS

### 2.1 Ethical approval

The two elite skiers whose data are reported here provided written informed consent. The study conformed to the standards set by the *Declaration of Helsinki*, except for registration in a database. The study received approval from the local ethics committee of the Université Savoie Mont‐Blanc (no. 2022‐19‐CVBSA).

### 2.2 Participants

Two elite skiers, both Olympic medallists, participated in this study (Skier 1: 26 years, 179 cm, 79 kg; Skier 2: 28 years, 179 cm, 80 kg). Their International Ski Federation (FIS) points in slalom were 5.2 and 6.0, respectively.

### 2.3 Protocol

This study was conducted as a part of a larger project examining neuromuscular responses in elite skiers (Alhammoud et al., 2022). The primary outcome measures related to joint kinematics do not overlap with previous neuromuscular function analysis.

Briefly, the skiers performed eight runs of slalom, with an average run duration of 28.36 ± 0.32 s and no disqualifying faults observed. All runs were timed using a starting gate and three photocells (two intermediate and one final). The course set‐up complied with FIS regulations (Table 1). The rotation time between runs was ∼20 min, including ∼9 min on a chairlift. The session lasted 132 min, preceded by an indoor warm‐up and ∼45–60 min of free skiing (Alhammoud et al., 2021a). The track was somewhat bumpy, and the snow conditions varied, being soft for the first four runs and then showcasing a compact snow layer for runs 5–8. Staff members regularly performed side slipping through the gates to limit the formation of micro‐reliefs between runs.

**TABLE 1:** Course setting.

Turn	Loaded foot	Turn direction	Tested leg	Cycle
*1*	*L*	*R*	*IL*	*1*
*2*	*R*	*L*	*OL*	
3	L	R	IL	2
4	R	L	OL	
5	L	R	IL	3
6	R‐double	L	OL	
7	L‐double	R	IL	4
8	R	L	OL	
9	L	R	IL	5
10	R	L	OL	
11	L‐delayed gate	R	IL	6
12	R	L	OL	
13	L	R	IL	7
14	R	L	OL	
15	L	R	IL	8
16	R	L	OL	
17	L	R	IL	10
18	R‐double	L	OL	
19	L‐double	R	IL	11
20	R	L	OL	
21	L	R	IL	12
22	R‐triple	L	OL	
23	L‐triple	R	IL	13
24	R‐triple	L	OL	
25	L	R	IL	14
26	R	L	OL	
27	L	R	IL	15
28	R	L	OL	
*29*	*L*	*R*	*IL*	*16*
*30*	*R*	*L*	*OL*	

*Note*: Course setting of the slalom training race. R: right, L: left, T: turn, IL: inside leg, OL: outside leg. Turns toward the left are shaded in grey. Cycles 1 and 16 (turns 1,2,29,30 in italic) were discarded from the analyses. The course set‐up included 32 gates, 10 m vertical spacing, 4 m offset, and figures (e.g., double, triple gates, delayed gate), 23.4% mean slope, 89 m vertical drop. Visibility was good with sunny weather. The temperature remained stable at 2°C during the session, with a slight increase to 3°C by the end. During the ski turn, a higher proportion of the forces applied between the skier and the ski is borne by the external ski (right leg for a left turn, left leg for a right turn). The loaded foot corresponds to the foot outside the turn.

### 2.4 Data acquisition

All data (3D acceleration and joint position) were collected with a Trigno Personal Monitor (TPM, Delsys, Natick, MA, USA, sampling frequency: 148 Hz) located on the skiers’ abdomen under the racing suit. Turn switches were determined from the 3D acceleration signal (Alhammoud et al., 2020). The resultant acceleration was computed (i.e., $\sqrt{x^{2}+y^{2}+z^{2}}$) and low‐pass filtered at 3 Hz. Then, the minima of the signal derivative were detected to identify the turn switches that were subsequently confirmed by visual inspection of the synchronized videos by the same investigator (M.A.). The elapsed time between two consecutive minima was computed as the turn time for each numbered gate.

**FIGURE 1:**
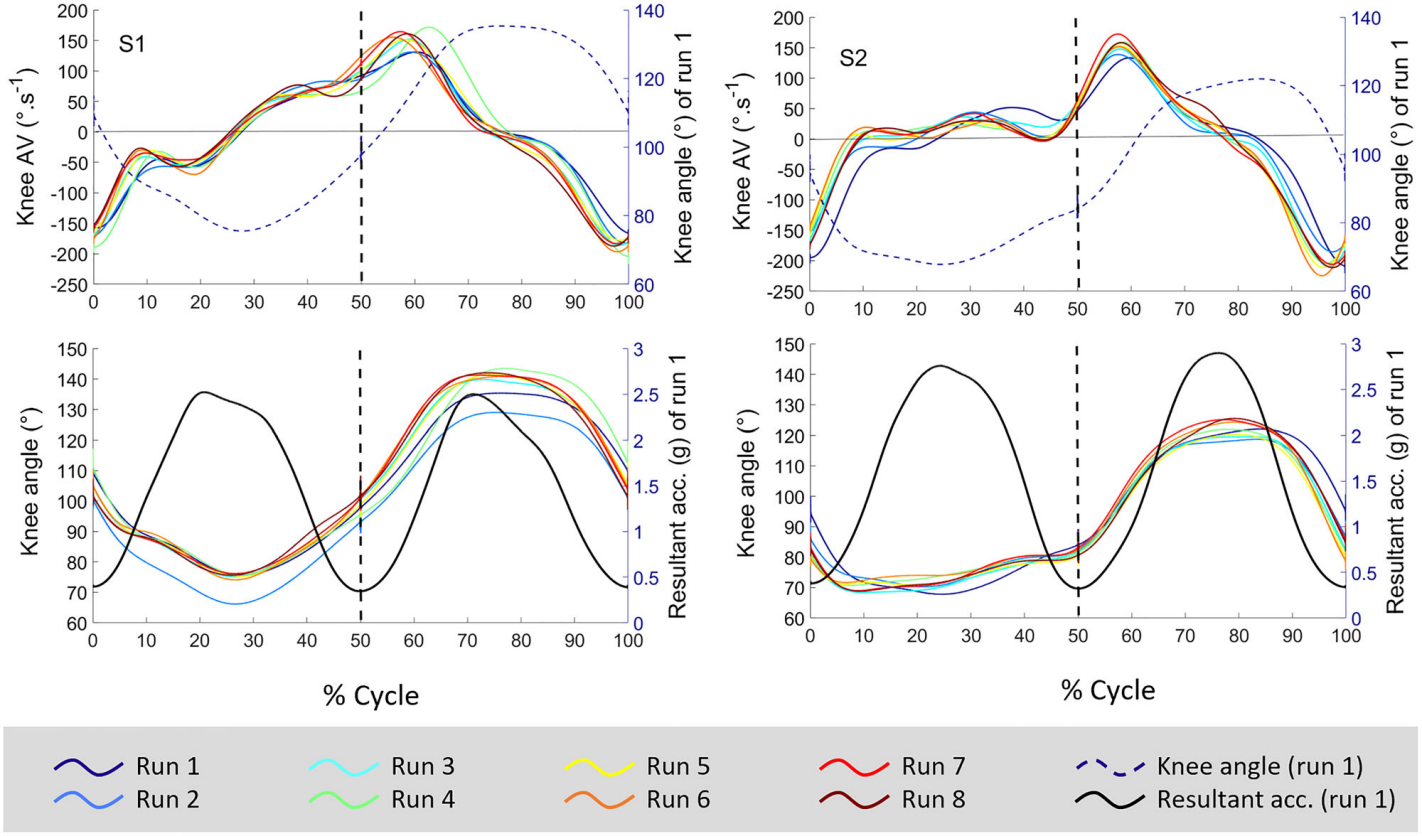
Individual patterns of the right knee angle and angular velocity across the time‐normalized ski cycle during eight runs of slalom. Data are presented separately for the two skiers (S1, left panel and S2, right panel). The upper panels represent the evolution of knee angular velocity (AV) across eight runs (14 cycles averaged per run), with the average knee angle of run 1 represented by the dotted line (right *y*‐axis). Lower panels represent the evolution of knee angle across eight runs (14 cycles averaged per run), along with the mean resultant acceleration of run 1 (right *y*‐axis, filtered with a lowpass Butterworth, cut‐off frequency at 1 Hz for the illustration) depicted by the black thick line. Each cycle is composed of a right turn (inside leg, 0%–50%) and a left turn (outside leg, 50%–100%). The turn switch is materialized by a dark dotted line at 50% of the cycle. Turn switches were determined from a triaxial accelerometer located on the skiers’ abdomen. Mean joint angle traces were calculated by normalizing each turn to the same number of data points. Due to unequal temporal lengths and slight variations, the turn duration was normalized to 100%. Then, to artificially reconstruct the ski cycle, angle–time traces during right and left turns were represented side by side, and time was once again normalized to 100% of the ski cycle, with 0%–50% representing the IL and 50%–100% the OL. This representation aligns with the fact that, in alpine skiing, the unit of interest is a double turn, namely the ski cycle.

The right leg's knee (KR) and hip (HR) angles were continuously monitored using electrogoniometers (SG 150, Biometrics Ltd, Newport, UK) (Alhammoud et al., 2020, 2021b) connected to the TPM via a Trigno Goniometer Adapter (Delsys, sampling frequency: 148 Hz). Signals were low‐pass filtered at 2.5 Hz and derived to compute the joint angular velocity as well as extension and flexion phases (Alhammoud et al., 2022). An example of mean knee angle and angular velocity traces (normalized by turn duration) is depicted in Figure 1.

A cycle was defined as a right (inside leg, IL) plus left (outside leg, OL) turn, separated by the edge changing phases (occurring between 0% and 10%, 40% and 60% and 90% and 100% of the cycle) (Nakazato et al., 2011). The first and last cycles (i.e., push‐off and deceleration phase) were discarded from the analyses, resulting in 14 IL + 14 OL per run per skier and a total of 448 signals (224 cycles). Outcome measures included the minimal and maximal angles, the amplitude of movement, the peak angular velocity (AV_peak_, °·s^−1^) during the joint flexion and extension phases, as well as run and turn time (ms).

### 2.5 Statistical analysis

Signal postprocessing was carried out using a custom routine (MATLAB 2017a, MathWorks, Natick, MA, USA). There were eight runs, 14 cycles, for sides among two participants which resulted in 448 rows of data for analysis. Outcome measures were analysed using SPSS Statistics v21.0 (IBM Corp., Armonk, NY, USA) via linear mixed models to evaluate the repeated run effect (eight runs), leg (inside or outside leg), and their interaction. For significant main effect, *post hoc* pairwise comparisons were performed after adjusting with the Bonferroni correction. Significance was defined as *P *< 0.05. Effect‐sizes were reported as partial eta‐squared (η^2^ ≥ 0.06 moderate effect, η^2^ ≥ 0.14 large effect). Data were also plotted as a function of run number with the slope of the linear regression being used as an indicator of the rate of change.

## 3 RESULTS

### 3.1 Performance

There was no effect of the run (*P* = 0.279) nor the interaction between run and photocell number (*P* = 0.342) on time (Table 2). The turn time was higher for IL than OL (mean leg difference [95%CI]:76 [58, 94] ms, *P *< 0.001, η^2^ = 0.18), but without effect of run (*P* = 1.000, η^2^ = 0.01) or interaction between run and leg (*P* = 0.691, η^2^ = 0.01) (Table 2).

**TABLE 2:** Performance and joint kinematics parameters.

			Run								*P*		
	Joint	Leg	1	2	3	4	5	6	7	8	Run	Leg	Int
Run time (s)			28.63 ± 0.17	28.51 ± 0.17	28.23 ± 0.17	27.93 ± 0.17	28.16 ± 0.17	28.5 ± 0.17	28.35 ± 0.17	28.59 ± 0.17	0.279	–	–
Turn time (ms)		IL	962 ± 64	951 ± 61	912 ± 61	912 ± 61	929 ± 61	916 ± 61	922 ± 61	948 ± 63	1.000	<0.001	0.691
		OL	886 ± 63	855 ± 61	872 ± 61	836 ± 61	831 ± 61	866 ± 61	845 ± 61	854 ± 64		IL > OL	
Amplitude (°)	HR		31 ± 3	34 ± 3	33 ± 3	33 ± 3	34 ± 3	36 ± 3	36 ± 3	35 ± 3	0.913	—	—
	KR		61 ± 4	64 ± 4	64 ± 4	66 ± 4	63 ± 4	67 ± 4	68 ± 4	70 ± 4	0.897	—	—
Min Angle (°)	HR	IL	100 ± 3	99 ± 2	99 ± 2	99 ± 2	98 ± 2	96 ± 2	98 ± 2	95 ± 3	0.842	<0.001	0.151
		OL	107 ± 3	106 ± 2	106 ± 2	103 ± 2	103 ± 2	102 ± 2	104 ± 2	100 ± 3		OL > IL	
	KR	IL	69 ± 2	68 ± 2	68 ± 2	70 ± 2	70 ± 2	69 ± 2	69 ± 2	68 ± 2	0.999	<0.001	0.705
		OL	89 ± 2	89 ± 2	88 ± 2	86 ± 2	87 ± 2	87 ± 2	88 ± 2	87 ± 2		OL > IL	

*Note*: Estimate ± standard error (SE) for the outside and inside leg (OL, IL), angle of the right knee and hip (KR, HR); along with the effect of run, leg, and the interaction effect between run and leg (Int). Of note, turn time was always longer on IL compared to OL (range: +40–96 ms) due to the slope configuration, with sloped terrain orientated on the right. Therefore, the right turns, when the left foot was loaded, were slightly prolonged as solely the right leg was instrumented. The coefficient of variation for run time was 1.1%.

### 3.2 Angles

**FIGURE 2:**
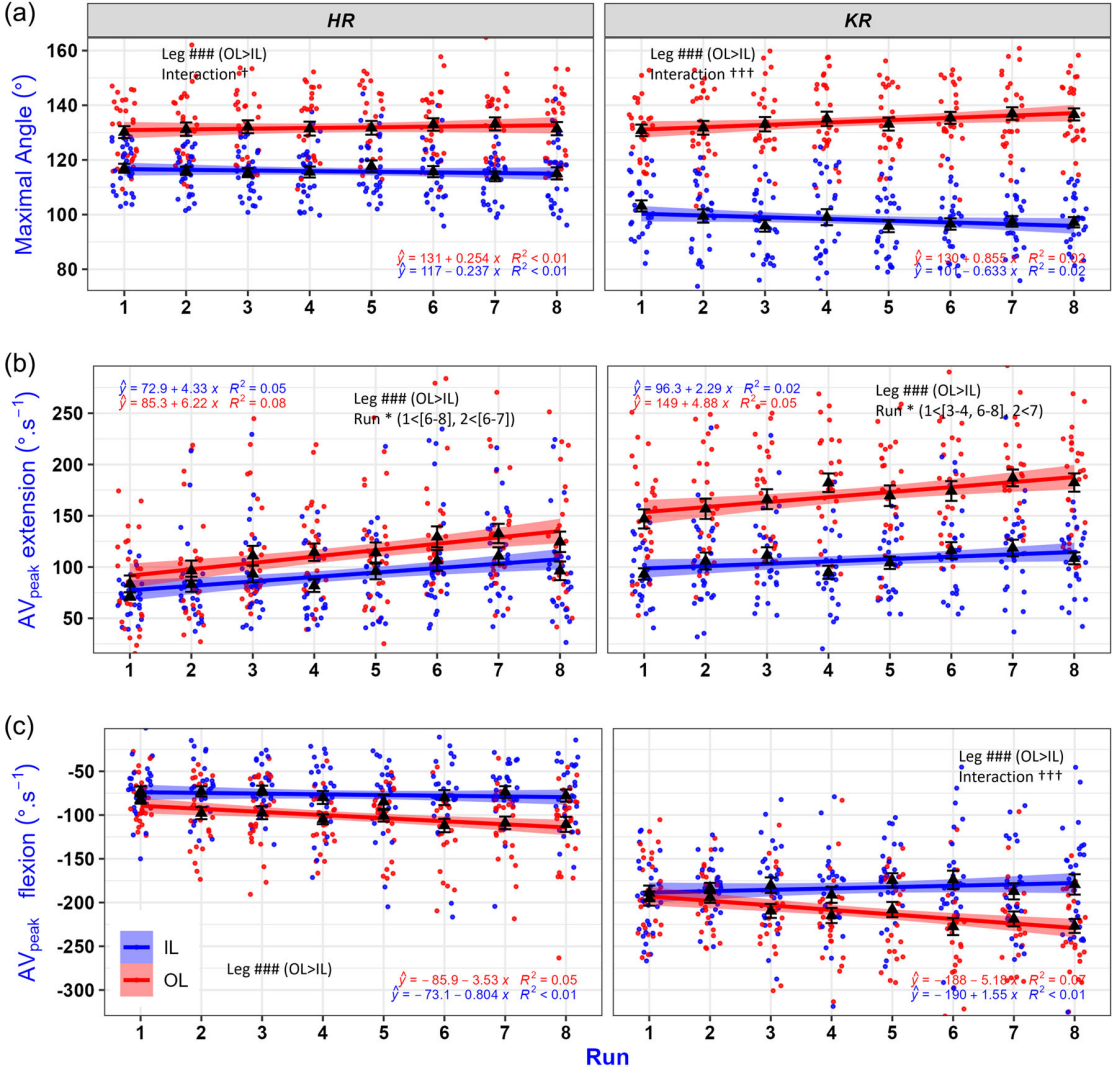
Kinematic adaptations of the right hip (HR, left panels) and knee (KR, right panels) for the inside (IL, blue) and outside (OL, red) leg across eight runs of slalom (average of the two skiers). The analysis primarily focused on the effects of repeated runs rather than individual participants. Consequently, turns from both participants are pooled together. The average of these turns is plotted for each run as a black triangle; however, individual data (448 turns) were retained in the linear mixed models. Mean value (▲) and regression line ±SE; each dot (●) represents one turn. # Effect of leg, * effect of run, † interaction effect between run and leg (* † *P *< 0.05, ### ††† *P *< 0.001). (a) Maximal angle (°, full extension = 180°). (b) Peak angular velocity (AV_peak_, °·s^−1^) during the joint extension phase. (c) Maximal angular velocity (°·s^−1^) during the joint flexion phase.

There was no effect of run on amplitude for HR or KR angles (*P* ≥ 0.897, η^2^ ≤ 0.05). The minimal angle was lower on IL than OL for both HR (−6 [−7, −5]°, η^2^ = 0.51) and KR (−19 [−20, −17]°, η^2^ = 0.78) (both *P *< 0.001), but without effects of run or interaction between run and leg (*P *> 0.151, η^2^≤0.05, Table 2). The maximal angle was higher on OL than IL for both HR (+16 [15, 17]°, η^2^ = 0.79) and KR (+36 [35, 37]°, η^2^ = 0.92) (both *P *< 0.001, Figure 2), without effects of run (*P* ≥ 0.964, η^2^ ≤ 0.02). There was an interaction between run and leg for both HR (*P* = 0.047, η^2^ = 0.04) and KR (*P *< 0.001, η^2^ = 0.12). The interaction effect was characterized by a positive coefficient of the linear regression (i.e., an increase across runs) on OL for both the HR (+0.254°·run^−1^) and KR (+0.855°·run^−1^), whereas this coefficient was negative (i.e., a decrease) on IL (HR −0.237°·run^−1^, KR −0.633°·run^−1^, Figure 2).

### 3.3 Joint angular velocity

The AV_peak_ in extension was higher on OL than IL for both HR and KR (*P *< 0.001, η^2^ ≥ 0.18, Figure 2). There was an effect of run for both HR and KR (*P* ≤ 0.028, η^2^ ≥ 0.11). *Post hoc* analyses showed that AV_peak_ in extension of HR was lower in run 1 than runs 6–8, and in run 2 than runs 6–7 (*P* ≤ 0.032). Similarly, the AV_peak_ in extension of KR was lower in run 1 than runs 3–4 and runs 6–8, and lower in run 2 than run 7 (*P* ≤ 0.043). The was no interaction between the effects of leg and run (*P* ≥ 0.239, η^2^ ≤ 0.05).

The AV_peak_ in flexion was faster (i.e., lower negative value) on OL compared to IL for both HR (−25 [−31, −19]°·s^−1^, η^2^ = 0.29) and KR (−28 [−34, −22]°·s^−1^, η^2^ = 0.27) (*P *< 0.001, Figure 2). There was no effect of run for HR or KR (*P* ≥ 0.264, η^2^ ≤ 0.07), and there was no interaction between run and leg for HR (*P* = 0.395, η^2^ = 0.04). However, there was an interaction effect for KR (*P *< 0.001, η^2^ = 0.13), with *post hoc* analyses showing no effects of run on IL (all *P* ≥ 0.132) but lower values on OL (i.e., faster AV in flexion) in the later runs compared to the initial ones (6–8 > 1; 6, 8 > 2) (*P* ≤ 0.025). Thus, while there was no leg effect on runs 1–2 (*P* = 0.232), a difference appeared from run 3 onwards (*P* ≤ 0.007). A linear regression showed a negative coefficient (i.e., increase in AV in flexion across runs, higher speed) on OL (−5.18°·s^−1^·run^−1^), whereas this coefficient was positive (i.e., decrease in AV in flexion across runs, slower speed) on IL (+1.55°·s^−1^·run^−1^).

## 4 DISCUSSION

Current data showed kinematic adaptations across eight runs, while turn and run time remained equivalent. This suggests that world‐class alpine skiers can reach similar performance with multiple kinematic patterns (notably larger leg dissociation), likely a response to both fatigue and slope evolution. Indeed, the increasing knee and hip angles represent an adaptation that tends to reduce muscular work by approaching a more extended posture on OL whilst IL bending increased.

### 4.1 Performance and position

There was an expected leg effect on all variables, along with an interaction between leg and run for both hip and knee maximal angles. The lower limb appeared more extended on OL (Figure 2a). Despite athletes typically facing difficulties at managing eccentric load (Gathercole et al., 2015a; Ruggiero et al., 2022) and sustaining a low position due to fatigue (Kiryu et al., 2011; Szmedra et al., 2001), our data showed that the small increase in the maximal angle on OL was accompanied by a more flexed IL. This suggests enhanced leg dissociation across runs repetition with the feet spreading apart, potentially in response to the groove created. Indeed, this technical evolution was corroborated by the coaches’ interviews, aiming to create more distance and altitude difference between the legs to avoid ‘turning on the IL’. The knee maximal angle increased on OL by +6 [−3, +14]° and decreased on IL by −6 [−14, +3]°, indicating a slightly more flexed position with small variation over time. In previous observations, intermediate but not experienced skiers adopted a more upright posture after repeated alpine ski turns within and among trials (5 min ski, 4000 m) (Kiryu et al., 2011). In our study, the two skiers did not adopt a more upright posture, suggesting that the influence of repeated runs on skiing performance is related to skiing level. Instead, they exhibited a fine‐tuning in their posture, possibly indicating more dissociation (i.e., more extended on OL and more flexed on IL, increasing bilateral leg difference from 28 [24, 31]° at run 1 to 39 [36, 43]° at run 8 for KR).

While completion time increased in collegiate skiers performing six training runs of 45 gates with shorter chairlift rotation (6 min) (White & Wells, 2015), our data showed no significant changes in intermediate and run time, nor turn time, across eight runs during a world‐class skier training session (Table 2). This highlights a marked performance stability in this population, along with the possibility of reaching the same performance with different kinematic responses. Previous studies showed that similar performance in giant slalom can be achieved by different turning strategies such as S‐ or Z‐shaped turns (Cross et al., 2021; Delhaye et al., 2020). The concept of adapting movement strategies is supported by reports of athletes adjusting their posture to keep the same countermovement jump performance despite fatigue (Gathercole et al., 2015a; Ruggiero et al., 2022). For example, elite snowboard cross‐athletes may maintain jump height after a lower‐body fatiguing protocol while lengthening the duration of the downward and upward movements (Gathercole et al., 2015b).

### 4.2 Joint angular velocity

The AV_peak_ in flexion for KR showed an interaction between leg and run, with a minor increase in IL (∼12°·s^−1^ from run 1 to 8, slower ‘eccentric’ velocity) and a marked decrease on OL (∼40°·s^−1^ from run 1 to 8, faster ‘eccentric’ velocity). The faster ‘eccentric’ velocity on OL could suggest a motor control strategy or a decreased ability of the muscles activated during lengthening movements to function as shock absorbers to decelerate knee flexion under high external loading (Vogt & Hoppeler, 2014), especially at the end of the carved turn. This aligns with previous studies showing that fatigue prolongs the eccentric phase of a countermovement jump (Gathercole et al., 2015a), a fundamental component of stretch‐shortening‐cycle exercise (Nicol et al., 2006) related to slalom (Frick & Schmidtbleicher, 1997; Vogt & Hoppeler, 2009, 2014). Conversely, the increase in AV_peak_ in extension with runs repetition indicates that the skiers extended their knee and hip faster (knee OL ∼+40/IL ∼+18°·s^−1^; hip OL ∼+50/IL ∼+35°·s^−1^ from run 1 to 8, faster ‘concentric’ velocities, Figures 1 and 2b).

The stability of macroscopic performance observed in elite athletes (Gathercole et al., 2015a; Ruggiero et al., 2022) is consistent with findings in skilled performers who can modulate their strategy to prevent exercise‐induced performance decline (Knicker et al., 2011). Indeed, skilled performers may exhibit greater movement variability to achieve consistent performance outcomes (Seifert et al., 2013). Current data indicate that world‐class skiers likely exhibit similar adaptability, supporting the concept of employing multiple movement strategies to achieve comparable performance (Cross et al., 2021), especially under fatigue conditions (Knicker et al., 2011).

### 4.3 Limitations and perspectives

Current data suggests that fatigue may manifest as an altered movement strategy rather than solely a lengthened run time. However, the current changes in SL may also partly reflect the changes in slope conditions, as deeper tracks and groove formation occur due to the limited gliding phase (Reid et al., 2020). While the high practicability of goniometers permitted an on‐field assessment in the current study, future investigations should consider three‐dimensional approaches (Martínez et al., 2019; Neuwirth et al., 2020). Therefore, the use of kinematics and kinetic variables, along with monitoring of the ski‐snow properties and video recording, appears warranted. To account for the small sample size, linear mixed models were deemed appropriate to retain the variability of the entire dataset, representing a preferred statistical method compared with traditional ANOVA. Despite the small sample size (*n* = 2), this novel approach provides valuable insights into kinematic adaptations in world‐class athletes.

### 4.4 Conclusion

Current data showed that the joint kinematic pattern of world‐class skiers evolves across the repetition of eight SL runs. However, macroscopic performance remains stable, showing that elite athletes may adopt multiple kinematic patterns (including a larger IL/OL dissociation) to reach the same performance. This adaptability is likely influenced by a combination of factors such as fatigue and ski‐snow interaction effects, including groove formation.

## AUTHOR CONTRIBUTIONS

Conception of the project: Marine Alhammoud, Christophe André Hautier, Sébastien Racinais, Baptiste Morel. Data acquisition: Marine Alhammoud, Sébastien Racinais. Data analyses: Marine Alhammoud, Robin Trama, Clint Hansen, Abdulaziz Farooq, Baptiste Morel. Data interpretation: Marine Alhammoud, Christophe André Hautier, Olivier Girard, Sébastien Racinais, Clint Hansen, Frédéric Meyer, Jérémy Coint, Thibaut Trameau, Loïc Brun, Baptiste Morel. Manuscript correction: all authors. All authors have read and approved the final version of this manuscript and agree to be accountable for all aspects of the work in ensuring that questions related to the accuracy or integrity of any part of the work are appropriately investigated and resolved. All persons designated as authors qualify for authorship, and all those who qualify for authorship are listed.

## CONFLICT OF INTEREST

None declared.

## FUNDING INFORMATION

None.

## Data Availability

All individual data for all participants (i.e., skiers 1 and 2) for each recording (i.e., run 1–8) are provided in Figure 2. Details can be obtained from the corresponding author upon reasonable request.
